# Efficacy and safety of thalidomide in children with monogenic autoinflammatory diseases: a single-center, real-world-evidence study

**DOI:** 10.1186/s12969-023-00881-0

**Published:** 2023-10-17

**Authors:** Caihui Zhang, Zhongxun Yu, Sihao Gao, Mingsheng Ma, Lijuan Gou, Changyan Wang, Lin Wang, Ji Li, Linqing Zhong, Yu Zhou, Wei Wang, Hongmei Song

**Affiliations:** Department of Pediatrics, Peking Union Medical College Hospital, Chinese Academy of Medical Sciences & Peking Union Medical College, No. 1 Shuaifuyuan Wangfujing Dongcheng District, Beijing, 100730 China

**Keywords:** Thalidomide, Monogenic autoinflammatory disease, Efficacy, Safety

## Abstract

**Background:**

Monogenic autoinflammatory diseases (AIDs) are rare inflammatory diseases caused by genetic variants. The pathogenesis is complex and treatment options are limited. This study aimed to describe the safety and efficacy of thalidomide in the treatment of monogenic AIDs.

**Methods:**

This was a single-center, single-arm, real-world study. From September 2016 to August 2021, patients with monogenic AIDs who met the inclusion and exclusion criteria were given thalidomide for 12 months. There was a 3-month run-in period before dosing. The efficacy and adverse events were evaluated and recorded every 3 months. After 3 and 12 months of thalidomide treatment, clinical manifestations, disease activity score, inflammatory markers, and background medication adjustments were compared with baseline for efficacy analyses.

**Results:**

A total of 16 patients entered this study, including 3 with Aicardi-Goutières syndrome (AGS), 4 Blau syndrome, 2 chronic infantile neurologic cutaneous articular syndrome (CINCA), 2 A20 haploinsufficiency (HA20), 1 adenosine deaminase 2 deficiency(DADA2), 1 familial Mediterranean fever (FMF),1 tumor necrosis factor (TNF) receptor-associated periodic syndrome (TRAPS), 1 PLCγ2-associated antibody deficiency and immune dysregulation (PLAID), and 1 stimulator of interferon genes-associated vasculopathy with onset in infancy(SAVI). The efficacy rate in the 16 patients after 3-month and 12-month thalidomide treatment in patients was 56.3%. Twelve patients completed the study, the fever improved in all of them, rash improved in 7 patients, and 5 patients stopped using glucocorticoids or other immunosuppressive agents. C-reactive protein was normal in 8 patients and erythrocyte sedimentation rate was normal in 11 patients. Anorexia and nausea occurred in 2 cases, with no other reported drug-related adverse reactions.

**Conclusion:**

The largest cohort of monogenic AIDs with the treatment of thalidomide demonstrated that thalidomide can help reduce disease activity and inflammation, reduce the dosage of glucocorticoids, and improve clinical outcomes. Thalidomide is relatively safe in monogenic AIDs.

**Supplementary Information:**

The online version contains supplementary material available at 10.1186/s12969-023-00881-0.

## Background

Monogenic autoinflammatory diseases (AIDs) are a group of genetic disorders caused by defects or dysregulations of the innate immune system that lead to continuous or recurrent inflammation. Currently, there are three main subgroups: type 1 interferonopathies, defects affecting the inflammasome, and non-inflammasome-related conditions [1, 2]. Monogenic AIDs usually present with constitutional symptoms like fever and inflammation-related clinical manifestations of multiple organ systems, including but not limited to the skin, joints, eyes, ears, gastrointestinal tract, respiratory tract, central nervous system and cardiovascular system [2–4]. While several medications have proven partially effective in some conditions, for example, colchicine for familial Mediterranean fever (FMF), interleukin 1 inhibitors for tumor necrosis factor (TNF) receptor-associated periodic syndrome (TRAPS), JAK inhibitors for several type 1 interferonopathies, glucocorticoids and conventional immunosuppressive agents for Blau syndrome and pyoderma gangrenosum and acne syndrome [5–7], there is no universal therapeutic agent for all kinds of AIDs, and these conditions are usually steroid-dependent and relapse once the dose is tapered. Another dilemma for monogenic AID patients in China is that some effective targeted biological agents such as canakinumab have not been marketed in China, and the drugs used in off label are also unaffordable. On this background, we turned our attention to a relatively old immunomodulatory drug, thalidomide. Previous studies have shown that thalidomide regulates signaling pathways through the NFκB-IRF4 pathway and AMPK/mTOR pathway, having anti-inflammatory, immunoregulatory and anti-angiogenic effects in cancer and autoimmune diseases [8], making it therapeutically useful in cancer, systemic lupus erythematosus, and Crohn's disease [9, 10]. Although short-term use of thalidomide has been seen in some isolated case reports of monogenic AIDs, data on long-term use in more pediatric monogenic AIDs patients are lacking. This study was dedicated to exploring the efficacy and safety of the long-term use of thalidomide in a pediatric monogenic AIDs series, in search of more options for the treatment of these diseases. We report the clinical and laboratory data of patients with monogenic AIDs who were treated with thalidomide and analyze its efficacy and adverse effects. The good outcomes provide another option for the treatment of AIDs.

## Materials and methods

### Patients

The inclusion criteria for this real-world-evidence study were as follows: (1) Children aged 0–18 with a genetic diagnosis of monogenic AID who were seen at the Department of Pediatrics, Peking Union Medical College Hospital between September 2016 and August 2021; (2) Active inflammation that could not be fully controlled by low-dose glucocorticoids and/or traditional immunosuppressive agents; (3) Patients and their legal guardians were willing to participate in this study. Exclusion criteria: (1) Liver dysfunction; (2) Neuropathy; (3) Allergy to thalidomide. All patients who completed the 3-month run-in period without altering their background treatment regimen were given thalidomide for 12 months. Patients could quit this study for any reason. All baseline (start of thalidomide) characteristics were stored and retrieved from the electrical medical record (EMR) system. A patient would be withdrawn from the study in case of (1) Discontinuation of thalidomide; (2) Addition of other drugs; (3) Increasing the dosage of an original drugs during thalidomide treatment. Discontinuation of treatment and replacement of other medications were permitted if patients had drug-related side effects (e.g., peripheral neuropathy) or poor efficacy during the study period.

### Treatment

The goal therapeutic dose was 1 to 2 mg per kilograms in children, with a maximal dose of 50 mg. For the convenience of medication, according to the patient weight and tolerability, patients took one-half tablet (12.5mg) to two tablets (50mg) every night. During the study period, if moderate to severe nausea, vomiting, drowsiness, hepatic impairment or other adverse events occurred, or the disease itself progressed or worsened, the research pediatricians could adjust the treatment regimen at any time to mitigate risks, and the subjects could also voluntarily withdraw from the study.

### Efficacy evaluation

**Fig. 1:**
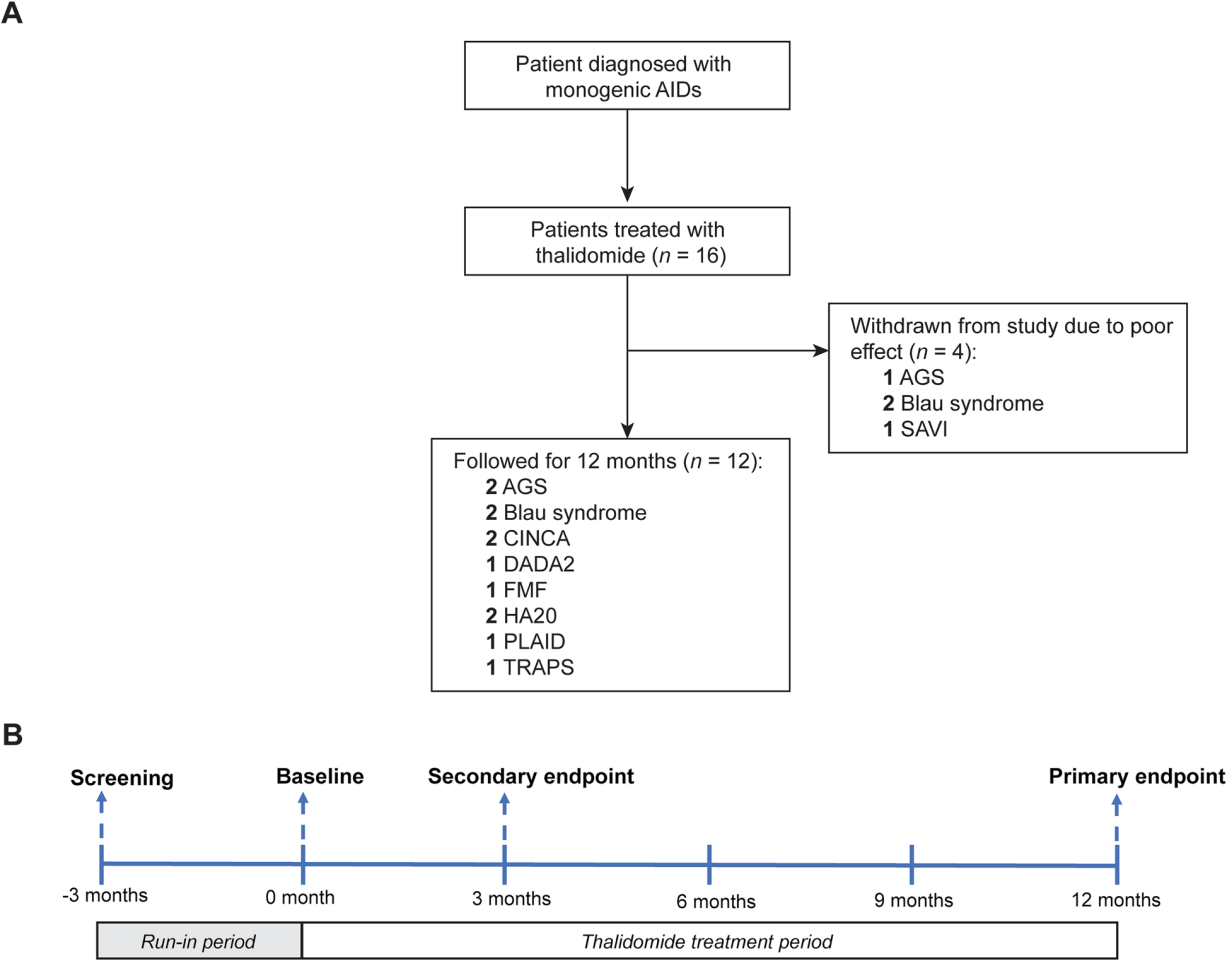
Flow diagram and timeline of the study. **A** Flow diagram for the selection of the patients in the study. **B** Timeline of treatment and data collection during the study. The numbers represent months since the baseline visit (0 month). -3 months refers to the observation period lasting 3 months before baseline

Due to the diversity of diseases included in the AIDs, there is no unified definition of clinical remission for all of them. Several scoring systems or clinical evaluation methods have been developed and used in several diseases, including Aicardi-Goutières syndrome (AGS), FMF, TRAPS, chronic infantile neurologic cutaneous articular syndrome (CINCA), A20 haploinsufficiency (HA20), Blau syndrome and stimulator of interferon genes-associated vasculopathy with onset in infancy (SAVI) (Additional file 1). To uniformly evaluate the efficacy of thalidomide in all monogenic AIDs, we comprehensively evaluated the following three aspects: (1) Improvement of disease activity score for diseases with a scoring system [such as Auto-Inflammatory Diseases Activity Index (AIDAI) for FMF and CINCA] or improvement of clinical manifestations for diseases without a scoring system; (2) Reduction to normalization of inflammatory markers (C-reactive protein (CRP) ≤ 8 and Erythrocyte Sedimentation Rate (ESR) ≤ 20); (3) Reduction or discontinuation of glucocorticoids, immunosuppressive agents or biologics. Thalidomide was considered effective if two or more of the above three items were met and the remaining item did not deteriorate. Efficacy evaluations were performed every 3 months and recorded in the EMR system. The primary endpoint was set at 12 months for long-term efficacy analysis and the secondary endpoint was set at 3 months for evaluating short-term efficacy. The above assessments were recorded every 3 months (Fig. 1).

### Safety evaluation

At each follow-up visit, patients underwent laboratory tests such as complete blood panel, serum biochemistry including liver function test and renal function test and electrocardiography as needed. Adverse events (AEs) were monitored in all participants throughout the treatment period. The severity of an AE and its association with thalidomide was assessed by the research pediatricians. Special attention was paid to gastrointestinal adverse events, neurological dysfunctions and hepatic impairment.

### Statistical analysis

Continuous (given as mean and standard deviation or median and quartiles) and categorical variables (frequency and percentages) were analyzed by GraphPad Prism 9. *P* values for comparisons between groups were obtained from paired nonparametric tests, *P* < 0.05 being statistically significant. Baseline characteristics and safety data are summarized from patients who took at least one dose of thalidomide. For efficacy assessment, especially for continuous variables, comparisons between the primary endpoints and baseline were performed in patients who completed the 1-year study. For the categorical variable of whether thalidomide was effective, patients who terminated treatment before to primary endpoint were regarded as non-responders and were imputed as noneffective at the primary endpoint.

## Results

### Baseline characteristics

A total of 16 patients entered this study. Their baseline characteristics are described in Table 1 and Additional file 1. There were 8 male patients and 8 female patients with an average age of 11.08 (IQR: 5.32–11.90) years old when starting thalidomide treatment, and the average course of disease was 6.20 ± 3.91 years. The age of disease onset was 1.0 (IQR: 0.33–4.73) years, the age at diagnosis was 7.25 (IQR:4.42–11.08) years, and the time from onset to diagnosis was 3.59 (IQR:1.38–7.0) years.

**Table 1:** Baseline characteristics of 16 patients with monogenic autoinflammatory diseases treated with thalidomide

**Characteristics (n, %)**	**AGS**	**Blau syndrome**	**CINCA**	**DADA2**	**FMF**	**HA20**	**PLAID**	**SAVI**	**TRAPS**	**Total**
**Num**	3 (18.75)	4 (25.0)	2 (12.50)	1 (6.25)	1 (6.25)	2 (12.50)	1 (6.25)	1 (6.25)	1 (6.25)	16 (100.0)
**Gender**										
Female	2 (25.0)	2 (25.0)	1 (12.50)	1 (12.50)	0	1 (12.50)	1 (12.50)	0	0	8 (50.0)
Male	1 (12.50)	2 (25.0)	1 (12.50)	0	1 (12.50)	1 (12.50)	0	1 (12.50)	1(12.50)	8 (50.0)
**Clinical manifestations**										
Fever	0	1 (12.50)	1 (12.50)	1 (12.50)	0	2 (25.0)	1 (12.50)	1 (12.50)	1 (12.50)	8 (50.0)
Rash	2 (16.67)	4 (33.33)	2 (16.67)	1 (8.33)	0	1 (8.33)	1 (8.33)	1 (8.33)	0	12 (75.0)
Growth or intellectual disability	1 (16.67)	2 (33.33)	2 (33.33)	0	0	0	0	1 (16.67)	0	6 (37.50)
Gastrointestinal involvement	1 (16.67)	0	1 (16.67)	1 (16.67)	1 (16.67)	1 (16.67)	1 (16.67)	0	0	6 (37.50)
Respiratory involvement	2 (28.57)	0	2 (28.57)	0	0	1 (14.29)	1 (14.29)	1 (14.29)	0	7 (43.75)
Cardiovascular involvement	1 (14.29)	3 (42.86)	2 (28.57)	0	0	0	1 (14.29)	0	0	7(43. 75)
Joint involvement	1 (33.33)	2 (66.67)	0	0	0	0	0	0	0	3 (18.75)
Eye or ear involvement	1 (14.29)	4 (57.14)	2 (28.57)	0	0	0	0	0	0	7 (43. 75)
Hematologic involvement	2 (28.57)	1 (14.29)	2 (28.57)	1 (14.29)	0	0	1 (14.29)	0	0	7 (43. 75)
Neurologic involvement	1 (20.0)	1 (20.0)	2 (40.0)	1 (20.0)	0	0	0	0	0	5 (31.25)
Hypothyroidism	1(50.0)	0	0	0	0	1(50.0)	0	0	0	2 (12.50)
Autoantibody positivity	2 (33.33)	1 (16.67)	0	0	0	1 (16.67)	1 (16.67)	1 (16.67)	0	6 (37.50)
Elevated CRP	0	3 (27.27)	2 (18.18)	1 (9.09)	1 (9.09)	1 (9.09)	1 (9.09)	1 (9.09)	1 (9.09)	11 (68.75)
Elevated ESR	2 (22.22)	2 (22.22)	1 (11.11)	1 (11.11)	0	1 (11.11)	0	1 (11.11)	1 (11.11)	9 (56.25)
**Treatment before the addition of thalidomide**										
Glucocorticoids	3 (27.27)	2 (18.18)	2 (18.18)	1 (9.09)	1 (9.09)	0	1 (9.09)	1 (9.09)	0	11 (68.75)
Immunosuppressive agents	3 (25.0)	3 (25.0)	1 (8.33)	1 (8.33)	1 (8.33)	1 (8.33)	0	1 (8.33)	1 (8.33)	12 (75.0)
Janus Kinase Inhibitors	3 (75.0)	0	0	0	0	0	0	1 (25.0)	0	4 (25.0)
MTX	0	3 (60.0)	1 (20.0)	1 (20.0)	0	0	0	0	0	5 (31.25)
Others	0	0	0	0	1^a^ (33.33)	1^b^ (33.33)	0	0	1^a^ (33.3)	3 (18.75)
Biologics	0	1 (50.0)^c^	0	1 (50.0)^d^	0	0	0	0	0	2 (12.50)

n (%) represents the number of positive cases in each disease category (number of positive cases in each disease category/total number of positive cases × 100%)

*DADA2* adenosine deaminase 2 deficiency, *PLAID* PLCγ2-associated antibody deficiency and immune dysregulation, *MTX* Methotrexate

^a^Colchicine

^b^Hydroxychloroquine

^c^Etanercept

^d^Tocilizumab

The patients shared some common characteristics, particularly recurrent fevers (8, 50.0%) and typical rash (12, 75.0%) depending on the subtype of monogenic AIDs. Various degrees of growth or intellectual disability were presented in 6 patients (37.50%). Hematological involvement was observed in 7 patients including 1 patient with thrombocytopenia and 6 patients diagnosed with anemia (Additional file 1). Regarding inflammatory indicators, eleven patients (68.75%) had increased CRP at the time of thalidomide add-on, and nine patients (56.25%) had elevated ESR. Before thalidomide treatment, 12 patients (75.0%) were taking immunosuppressive agents, 11 patients (68.75%) were taking glucocorticoids, and 2 patients (12.50%) were under treatment with biologics.

Four patients discontinued thalidomide gradually after reaching 3 months of treatment due to the inability to reduce lower inflammatory indicators to the normal range, failure to improve clinical symptoms or disease-free activities, or worsening of symptoms after discontinuation of glucocorticoids (Table 2).

**Table 2:** Evaluation of disease activity score or clinical symptoms in 16 patients

**No**	**Diagnosis**	**3 months before**	**Baseline**	**3 months**	**6 months**	**9 months**	**12 months**
1	AGS^a^	3	3	3	3	3	3
2	AGS^a^	1.26	3	3	3	3	3
3	AGS^a^	0.2	0.2	0	0	-	-
4	Blau syndrome^b^	0	0	0	0	0	0
5	Blau syndrome^b^	6	12	12	-	-	-
6	Blau syndrome^b^	0	0	0	0	0	0
7	Blau syndrome^b^	30	30	12	4	-	-
8	CINCA^c^	35	28	10	8	2	4
9	CINCA^c^	7	7	14	5	0	0
10	DADA2	Persistent fever, rash, oral ulcers and celialgia	Persistent fever, rash, oral ulcers and celialgia	Reduced fever frequency	Fever and celialgia improved	Few ulcers and rashes remained	Disappearance of rash
11	FMF^c^	15	15	0	0	0	0
12	HA20^d^	Mild	Mild	Disease-free activity	Disease-free activity	Disease-free activity	Disease-free activity
13	HA20^d^	Moderate	Moderate	Mild	Disease-free activity	Moderate	Disease-free activity
14	PLAID	Recurrent fevers and rash	Recurrent fevers and rash	Intermittent fever, rash and arthralgia	Intermittent fever, rash and arthralgia	Occasional fever and rash	Occasional fever and rash
15	SAVI^e^	3	3	1	2	2	-
16	TRAPS^c^	15	15	15	10	15	10

^a^Clinical score of AGS

^b^Number of active arthritis episodes

^c^AIDAI

^d^Disease severity of HA20

^e^Disease activity rating scale of SAVI

### Efficacy evaluation

#### Disease activity score or clinical manifestations

**Fig. 2:**
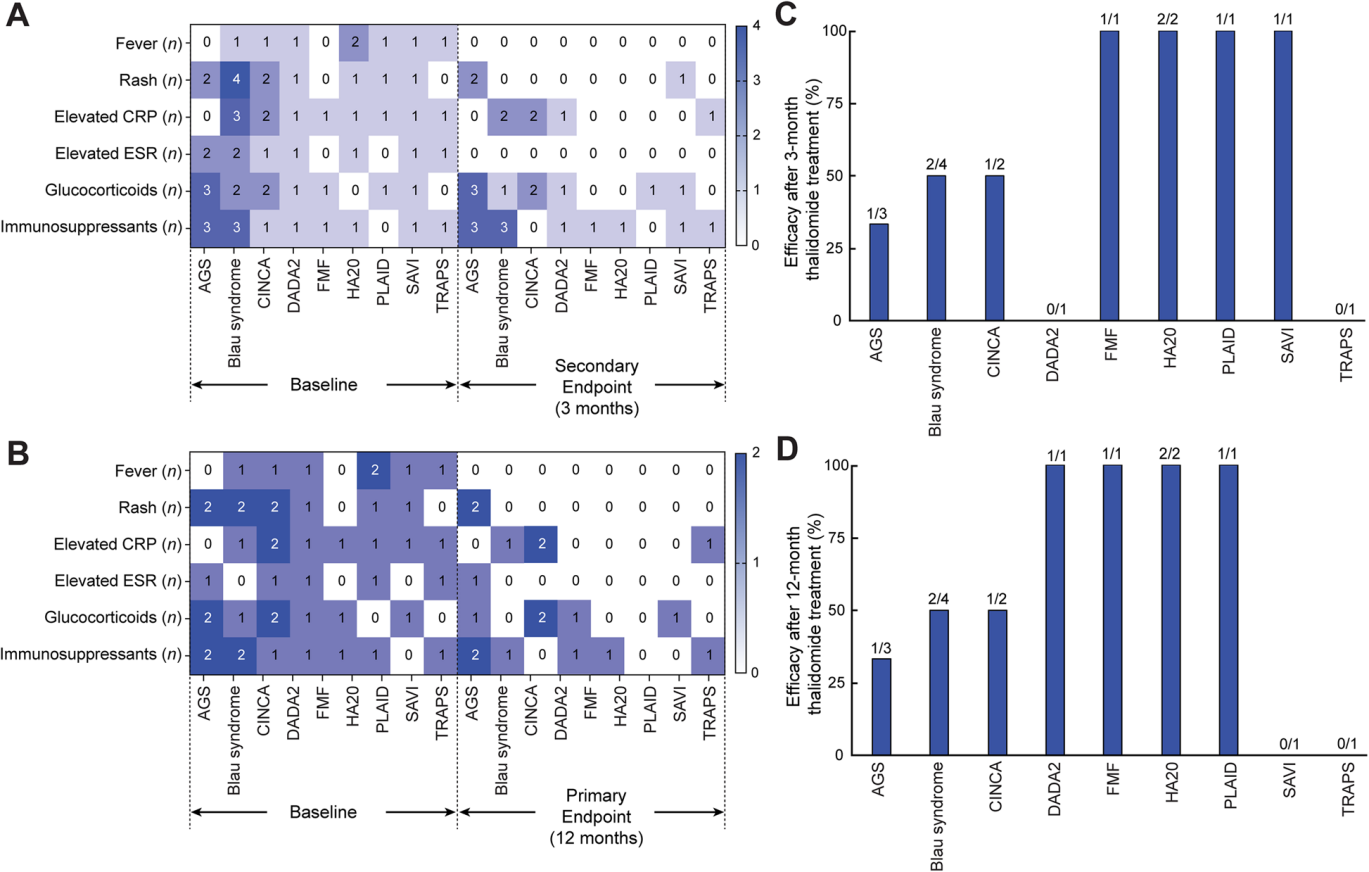
Efficacy evaluation in thalidomide. **A**–**B** Heatmaps showing the number of patients with different outcomes before and after thalidomide treatment. The horizontal axis represents the disease type, with baseline conditions on the left and endpoints on the right. The vertical axis represents clinical, laboratory, and treatment features. The color intensity reflects different numerical values, darker colors indicating higher values. **A** Baseline and 3 months after thalidomide treatment in 16 patients. **B** Baseline and 12 months after thalidomide treatment in 12 patients. **C** Efficacy rate after 3 months of thalidomide treatment. The number above the bar graph is the ratio of the patients with effective treatment out of all patients with different types of AIDs. **D** Efficacy rate after 12 months of thalidomide treatment. The number above the bar graph is the ratio of the patients with effective treatment out of all patients with different types of AIDs

After 3 months of treatment, 16 patients remained in this study, and the fever had resolved in all of them. The rash had disappeared in 9 (75.0%) patients (Fig. 2A). Among the 12 patients who took thalidomide for 12 months, seven patients had fever and nine patients had rash at baseline, the fever improved in all patients (100.0%) and rash improved in 7 (77.78%) patients with thalidomide treatment (Fig. 2B).

In terms of disease activity, 13 (81.25%) patients had disease activity comparable to baseline in the 3 months before thalidomide treatment (Table 2). Eleven patients (68.75%) had disease activity improvement (9, 81.82%) or continued disease-free activities (2, 18.18%) after 3 months of thalidomide treatment. Twelve patients were followed up for 1 year, including 10 patients (83.33%) with disease activity that improved from baseline (8, 80.0%) or remained absent (2, 20.0%).

Thalidomide was added to patients with AGS because of an elevated ESR (Case 1), recurrent rashes (Cases 1, 2), and the desire to withdraw glucocorticoid (Cases 1 and 2). In Case 1, the inflammatory markers decreased to normal, and glucocorticoid was successfully discontinued after treatment for 1 year. Case 2 had sustained ESR. Two patients with Blau syndrome (Cases 4 and 6) had persistent inactive arthritis after thalidomide treatment, of whom Case 4 discontinued glucocorticoid and remained on thalidomide alone by the end of follow-up. Furthermore, all four patients with Blau syndrome exhibited no activity of ophthalmia. The AIDAI decreased with the addition of thalidomide in 2 CINCA, 1 FMF and 1 TRAPS patients after the addition of thalidomide. After treatment with thalidomide, symptoms improved in patients with DADA2, HA20, and PLAID (Table 2).

#### Inflammatory markers

**Fig. 3:**
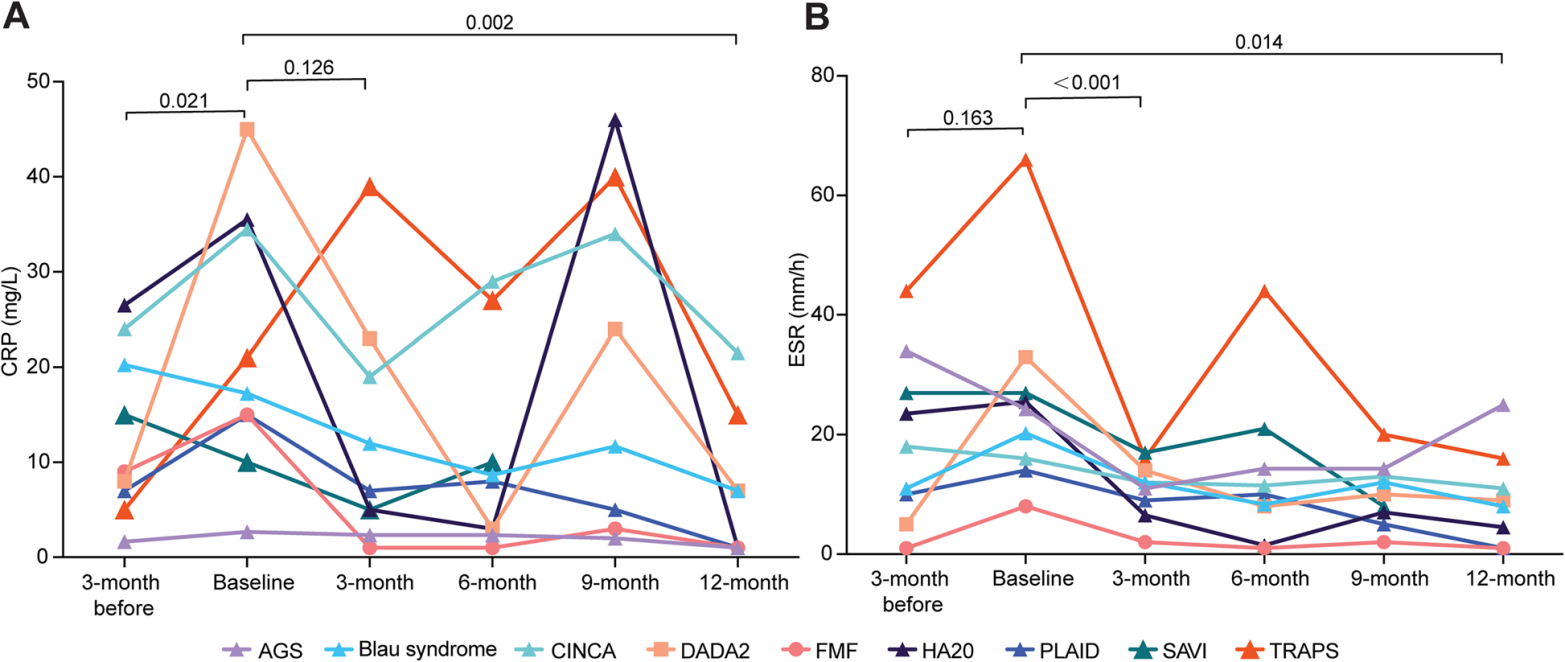
Time courses for CRP (**A**) and ESR (**B**) for each disease. The data were taken at baseline, 3 months before thalidomide and at months 3, 6, 9, and 12 on thalidomide. The color difference outlines the difference in disease types, and the numbers are* P* values from the paired test for each patient.* P* < 0.05 was considered statistically significant. Points on lines represent the mean values of the inflammatory index for each disease

Eleven patients had elevated CRP and nine had elevated ESR at baseline among the 16 patients treated with thalidomide for 3 months. Among them, the CRP levels in 10 patients (62.50%) and ESR levels in 16 patients (100.0%) reached the normal ranges (Fig. 2A). Of the 12 patients followed for 1 year, 8 patients (66.67%) had elevated CRP and 5 patients (41.67%) had elevated ESR at baseline. Eight patients (66.67%) had CRP values within the normal range at the end of follow-up (Fig. 2B), and the CRP of the three patients whose CRP remained abnormal at the end of follow-up decreased to more than 80% of the original values; ESR decreased to the normal range after 1 year of treatment in 11 patients (91.67%) (Figs. 2B and 3B).

The CRP levels of patients at baseline were significantly higher than those before thalidomide treatment (*P* = 0.021), and the ESR levels were also higher than those before thalidomide treatment, but this difference did not reach statistical significance (*P* = 0.163), which means that patients under the original treatment regimen might not have maintained the inflammatory indicators in the normal ranges. After 1 year of treatment with thalidomide, the values of CRP and ESR were decreased from baseline (*P* = 0.002, 0.014), indicating that thalidomide can effectively dampen the inflammatory status. Furthermore, the level of ESR after 3 months of thalidomide was significantly lower than at baseline (*P* < 0.001), while there was no significant change in CRP from baseline to 3 months (*P* = 0.126) (Fig. 3A, B and Table 3).

**Table 3:** Laboratory data before and after the treatment of thalidomide

**Inflammatory markers (median [interquartile range])**	**3 months before**	**Baseline**	**3 months**	**6 months**	**9 months**	**12 months**
**CRP (mg/L)**	9.0 (5.0, 15.0)	16.50 (3.75, 27.75)	5.50 (3.25, 21.23)	5.0 (1.0, 13.0)	6.0 (2.50,28.0)	1.0 (1.0,14.50)
**ESR (mm/h)**	14.0 (8.0, 30.0)	24.0 (8.0, 36.0)	9.50 (5.75, 16.0)	8.0 (4.0, 19.0)	8.0 (5.0,19.0)	7.0 (2.75,15.8)

CRP and ESR values were recorded at baseline, 3 months before thalidomide and at 3, 6, 9, and 12 months on thalidomide. Data are median (interquartile range)

#### Withdrawal of other medications

Twelve patients (75.0%) received immunosuppressive agents at baseline (Table 1) and 1 (8.3%) of whom discontinued MTX after 3 months of treatment. Eleven patients (68.8%) received treatment for glucocorticoids before the screening period and 2 of whom discontinued glucocorticoids after 3 months of treatment with thalidomide (Fig. 2A). Among the 12 patients followed for 1 year, 3 patients (25.0%) discontinued glucocorticoids, 3 (25.0%) discontinued immunosuppressive agents, and 1 (8.3%) patient discontinued the biologic agent. Among them, 1 patient with Blau syndrome discontinued glucocorticoids and immunosuppressive agents simultaneously, and 3 patients (cases 4,12,13) were treated with thalidomide alone (Fig. 2B). Two patients (12.5%, cases 6 and 14) had their glucocorticoids or immunosuppressive agents reduced to low-dose maintenance therapy.

In summary, considering the clinical manifestations or disease activity scores, inflammation markers and background medication adjustments, the efficacy rate in 16 patients after 3 months and 12 month of thalidomide treatment in children with monogenic AIDs was 56.3% (Fig. 2C, D). Efficacy in DADA2, FMF, HA20 and PLAID after 12 months of thalidomide treatment was 100.0%. The efficacy in other groups of AIDs of thalidomide treatment for 3 and 12 months was shown in Fig. 2C and D.

### Safety evaluation

Two patients (12.50%, cases 5 and 12) experienced anorexia and nausea at a dose of 25 mg/d (1.0–1.5 mg/kg), which resolved after dosage reduction, and no obvious discomfort was reported after that. None of the patients had adverse events such as peripheral neuropathy and somnolence.

## Discussion

In the latest 2022 inborn errors of immunity (IEI) classification, the monogenic AIDs were divided into 3 major groups of 56 diseases [2]. The pathogenesis of these rare diseases involves activation of the innate immune system causing repeated or persistent inflammation with cytokine overproduction, especially tumour necrosis factor (TNF)-α, interleukin (IL)-1β, IL-6, IL-18, and interferon (IFN)-α [1]. Timely diagnosis and treatment are critical for a good prognosis. Unfortunately, most of these diseases are resistant to conventional immunosuppressive therapy. Previous studies have shown that thalidomide exerts anti-inflammatory, immunomodulatory, and angiostatic effects [11, 12]. Therefore, we attempted to add thalidomide to the current treatment in patients with monogenic AIDs. We report the largest cohort of monogenic AIDs treated with thalidomide in a pediatric population, who showed no intolerable side effects.

Thalidomide blocks the release of cytokines such as TNF-α, IFN-γ, IL-10, and IL-12, as well as cyclooxygenase 2 and the transcription factor nuclear factor NF-κB. Thalidomide has been considered to have potent broad-spectrum anti-inflammatory activity, which expands the possibilities for the treatment of inflammatory diseases [12], but evidence for the efficacy of thalidomide in AIDs is limited to sporadic case reports and series. Six of seven patients with DADA2 achieved persistent remission when the treatment of thalidomide [13]. Thalidomide can provide CINCA patients symptomatic relief of limited joint mobility, helping their weight gain and reducing pain associated with daily injections of biological agents [14]. Almost 10% of FMF patients are resistant or intolerant to colchicine treatment [15]. Thalidomide has persistently suppressed febrile attacks and acute phase responses in colchicine resistant FMF patients [16]. When two For HA20 patients were treated with thalidomide, both showed amelioration of gastrointestinal symptoms on a background of glucocorticoids or biological agents and eventually discontinued glucocorticoid therapy [17]. Our findings parallel the above data. Monogenic AIDs patients treated with thalidomide for 1 year in our study showed significant improvements in fever and rash, as well as control of inflammatory parameters, with decreased disease activity in most patients compared to baseline. Notably, some patients were even able to discontinue glucocorticoids and other immunosuppressive agents, suggesting the potential effectiveness of thalidomide in certain monogenic AIDs.

Previously, it was reported that during thalidomide treatment of patients with PLAID, glucocorticoids and MTX have been used concomitantly, which improved only some of the symptoms improved and decreased CRP transiently, and immunosuppressive agents were discontinued because of abnormal liver function [18]. Another patient with granulomatous disease did not see improvement of his skin lesions with the addition of thalidomide [19]. The clinical manifestations of the 1 PLAID patient in this study were milder than those reported in the literature (Additional file 2), and thalidomide was effective in combination with glucocorticoids, after which the glucocorticoids were tapered to a low dosage. Therefore, thalidomide treatment may be trialled in PLAID patients with relatively mild clinical symptoms. Thalidomide can inhibit the inflammatory response and improve clinical symptoms in patients with Blau syndrome, with few adverse reactions [20, 21]. The efficacy of thalidomide in Blau syndrome was 50% in this study (Fig. 2D), suggesting that more extensive inflammatory pathways or cytokines may be involved in the mechanism of Blau syndrome [22]. In addition, our cohort reported the efficacy of thalidomide after 1 year treatment of AGS, SAVI and TRAPS in clinical practice firstly [23]. Thalidomide was partially effective against AGS and ineffective against SAVI and TRAPS in this study, but at present, studies about the treatment of these disorders are few, and more research is needed to evaluate the efficacy and side effects of thalidomide.

As for other diseases with autoinflammatory features, thalidomide is efficacious at controlling inflammation in patients suffering from sideroblastic anemia, immunodeficiency, periodic fevers, and developmental delay [24]. Pyoderma gangrenosum, acne, and hidradenitis suppurativa syndrome have been seen in a patient who initially failed to respond to immunosuppressive treatment but responded to a combination of colchicine and thalidomide [25]. It has been reported that the clinical symptoms and inflammatory indicators of a Muckle-Wells syndrome patient improved after treatment with thalidomide [26]. All of the above suggest that thalidomide has a broad anti-inflammatory effect and may be worth trying for a variety of autoinflammatory diseases.

One major concern about the widespread use of thalidomide is its side effects. One adult patient with colchicine resistant FMF developed a lower extremity numbness during treatment with thalidomide, which disappeared despite continuing the drug [16]. In our study, the treatment with thalidomide was well tolerated overall, and no patient developed severe side effects during the treatment. Only 2 patients had drug-related side effects, which included nausea, fatigue, and anorexia. We took detailed medical history and physical examination with special attention to identify potential peripheral neuropathies, and none of the patients had the symptoms such as numbness, tingling, pain or tremor. Generally, the preference of one treatment over the other can therefore be based on the price and/or the country’s payment conditions of the drugs [27]. Despite the possible toxic effects on the peripheral nervous system, this drug has the advantage of a low cost and could be considered in developing countries where the use of biologic agents is limited for economic reasons [14].

Our study further confirmed that thalidomide could be considered a promising therapeutic option for monogenic AIDs not only for reducing disease flare-ups but also for reducing the inflammation index. Furthermore, thalidomide can help taper or discontinue glucocorticoids and immunosuppressive agents that have relatively strong adverse reactions. Thalidomide is cheaper than biologics such as anakinra and easier to purchase for Chinese patients. The successful application of thalidomide in monogenic AIDs paves the way for further study of this treatment option.

The first and main limitation of this study is its small sample size and retrospective design. Because the monogenic AIDs are rare, only patients treated with thalidomide were included. Further studies with larger patient cohorts are required to validate recent findings with thalidomide.

## Conclusion

This study evaluated the efficacy and safety of thalidomide in monogenic AIDs and is the largest study of thalidomide for the treatment of monogenic AIDs patients. Thalidomide can help alleviate clinical symptoms or disease activity in some patients, achieve remission of inflammatory indicators, and help the patient discontinue glucocorticoids or other immunosuppressive agents. The most common adverse reactions were gastrointestinal symptoms. During treatment, the dosage can start low and gradually increase to achieve therapeutic effects with the smallest dosage possible. Overall, data on the use of thalidomide in monogenic AIDs are scarce, so further studies are needed to evaluate its long-term efficacy and side effects.

### Supplementary Information

**Additional file 1.**

**Additional file 2.**

## Data Availability

The datasets are available from the authors upon reasonable request and with permission of the Institutional Review Board of Peking Union Medical College Hospital.

## Abbreviations

AIDs: Autoinflammatory diseases
FMF: Familial Mediterranean fever
TRAPS: Tumor necrosis factor receptor associated periodic fever
AGS: Aicardi-Goutières syndrome
CINCA: Chronic infantile neurologic cutaneous articular syndrome
HA20: A20 haploinsufficiency
SAVI: Stimulator of interferon genes-associated vasculopathy with onset in infancy
AIDAI: Auto-Inflammatory Diseases Activity Index
CRP: C-reactive protein
ESR: Erythrocyte Sedimentation Rate
EMR: Electrical medical record
AE: Adverse events
DADA2: Adenosine deaminase 2 deficiency
PLAID: PLCγ2-associated antibody deficiency and immune dysregulation
