# Supplementary material for: Efficacy and safety of thalidomide in children with monogenic autoinflammatory diseases: a single-center, real-world-evidence study
Source: Pediatr Rheumatol Online J. 2023 Oct 17;21:124. doi: 10.1186/s12969-023-00881-0 (PMC10583446; doi:10.1186/s12969-023-00881-0)
Supplement: Supplementary file 1 — Additional file 1. [file 12969_2023_881_MOESM1_ESM.docx]

**Additional information**

**Assessment of Disease activity**

**1.** **Aicardi-Goutières syndrome (AGS)**

For patients with AGS, the parents of the patients recorded the children’s related symptoms, including neurologic disability, crying, sleep disturbances, irritability, seizures, fever, and skin inflammation of the trunk, arms, and legs[1].

**Clinical scores**

| Category | Signs and Symptoms | Score |
| --- | --- | --- |
| Crying | Cries, but easily consolable | 0 |
|  | Excessive or high-pitched cry for > 2 min OR intermittently for < 10 min | 1 |
|  | Excessive or high-pitched cry for > 2 min AND intermittently for < 10 min | 2 |
|  | Excessive or high-pitched cry, not consolable (cries > 10 mins) | 3 |
| Sleep | Sleeps for > 3 hours continuously during night | 0 |
|  | Sleeps 2-3 hours continuously during the night | 1 |
|  | Sleeps 1-2 hours continuously during the night | 2 |
|  | Sleeps <1 hour continuously during the night | 3 |
| Irritability | No irritability | 0 |
|  | Consoling calms individual in < 6 min | 1 |
|  | Consoling calms individual in 6-15 minutes | 2 |
|  | Consoling calms individual in >15 minutes or not at all | 3 |
| Seizure | No convulsions or seizures | 0 |
|  | Experiences convulsions or seizures | 8 |
| Fever | No fever (temperature < 98.9 F) | 0 |
|  | Temperature greater than 99.1 F | 1 |
| Skin findings: body | No skin problems | 0 |
|  | Red patches which fade when pressed with fingers | 1 |
|  | Chronic discoloration | 2 |
| Skin findings: hands, face, ears | No skin problems | 0 |
|  | Red patches which fade when pressed with fingers | 1 |
|  | Red patched not fading when pressed with fingers | 2 |
|  | Chronic discoloration | 3 |

**2. Blau syndrome**

In patients with Blau syndrome, joint count = 0 was required for inactive arthritis, a standardization uveitis nomenclature grade of 0 for inactive anterior uveitis and a Nussenblatt scale of 0 for inactive vitritis[2]. Because none of the patients at baseline in our cohort had active uveitis and uveitis, the number of active joints was used as the disease activity score.

**3. Familial mediterranean fever (FMF), chronic infantile neurologic cutaneous and articular syndrome (CINCA)** **and tumour necrosis factor receptor-associated periodic syndrome (TRAPS)**

The Auto-Inflammatory Diseases Activity Index (AIDAI) was applied to evaluate the disease activity of FMF, CINCA and TRAPS[3, 4].

**Auto-Inflammatory Diseases Activity Index (AIDAI)**

| Days | Fever≥38 (100.4°F） | Overall symptoms | Abdominal pain | Nausea/vomiting | Diarrhea | Head-aches | Chest pin | Painful nodes | Arthralgia or Myalgia | Swelling of the joints | Eyes manifestations | Skin rash |
| --- | --- | --- | --- | --- | --- | --- | --- | --- | --- | --- | --- | --- |
|  |  |  |  |  |  |  |  |  |  |  |  |  |
| Scored as | 0/1 | 0/1/Yes/No | 0/1/Yes/No | 0/1/Yes/No | 0/1/Yes/No | 0/1/Yes/No | 0/1/Yes/No | 0/1/Yes/No | 0/1/Yes/No | 0/1/Yes/No | 0/1/Yes/No | 0/1/Yes/No |
| 1 |  |  |  |  |  |  |  |  |  |  |  |  |
| 2 |  |  |  |  |  |  |  |  |  |  |  |  |
| 3 |  |  |  |  |  |  |  |  |  |  |  |  |
| … |  |  |  |  |  |  |  |  |  |  |  |  |
| 31 |  |  |  |  |  |  |  |  |  |  |  |  |

Each line represents a day in a month.

**4. Haploinsufficiency of A20 (HA20)**

The disease severity of HA20 can be divided into three types according to the clinical manifestations. The mild type was defined as asymptomatic or minor phenotype (e.g., mild recurrent stomatitis and/or rash). The moderate type was defined as paroxysmal symptoms (e.g., recurrent fever and/or abdominal pain). The frequency of symptoms is once or more for at least 3 months. The severe type was defined as persistent inflammation (e.g., fever, central nerve lesion, vascular lesion, ocular lesion, intestinal lesion, and/or arthritis) sustained over 2 weeks[5].

**5. STING associated vasculitis with onset in infancy (SAVI)**

Disease activity rating scale of SAVI patients was evaluated according to the method described by Crow et al[6], and the sum of the scores of the 6 components yields the global disease score (range = 0-24).

| Fever during the last period:  0: No fever  1: Fever every month  2: Fever every week  3: Fever twice a week  4: Fever every day |
| --- |
| Erythematous lesions at visit:  0: No erythematous lesion  1: Erythematous lesions barely present  2: Erythematous lesions covering between 10% and 25% of body surface area  3: Erythematous lesions covering more than 25% of body surface area  4: Erythematous lesions covering more than 25% of body surface area and painful |
| Ulcer lesions at visit: The score is calculated for face, left hand, right hand, left foot, right foot, and others regions. The final ulcer score is the sum of the 6 components divided by 6.  0: No ulcers  1: Few ulcers, no oozing, no ischemia  2: Few ulcers, with some oozing, no ischemia  3: Few ulcers, with significant oozing, and/or any ischemia  4: Multiple ulcers, or with significant oozing and ischemia |
| Nail and hair lesions at visit:  Nail dystrophy:  0: No nail dystrophy  1: Mild and unilateral nail dystrophy  2: Severe or bilateral nail dystrophy  Hair lesions  0: No hair lesions  1: Mild thin hair  2: Very thin and breakable hair |
| Respiratory difficulties during the last period:  0: No dyspnea  1: Mild dyspnea, rapid breathing, but with no functional impact  2: Moderate dyspnea, rapid breathing, with mild functional impact  3: Severe dyspnea, rapid breathing, with severe functional impact (e.g. absence from school)  4: Severe dyspnea, rapid breathing, resulting in staying in bed, oxygen therapy |

**6. Others**

Other types of monogenic autoinflammatory diseases like adenosine deaminase 2 deficiency (DADA2), PLCγ2-associated antibody deficiency and immune dysregulation (PLAID) were evaluated according to their clinical manifestations.

**References：**

1. Vanderver A, Adang L, Gavazzi F, McDonald K, Helman G, Frank DB, et al. Janus Kinase Inhibition in the Aicardi-Goutières Syndrome. N Engl J Med. 2020;383(10):986-989. doi:10.1056/NEJMc2001362.

2. Wang L, Rosé CD, Foley KP, Anton J, Bader-Meunier B, Brissaud P, et al. S100A12 and S100A8/9 proteins are biomarkers of articular disease activity in Blau syndrome. Rheumatology (Oxford). 2018;57(7):1299-1304. doi:10.1093/rheumatology/key090.

3.Piram M, Frenkel J, Gattorno M, Ozen S, Lachmann HJ, Goldbach-Mansky R, et al. A preliminary score for the assessment of disease activity in hereditary recurrent fevers: results from the AIDAI (Auto-Inflammatory Diseases Activity Index) Consensus Conference. Ann Rheum Dis. 2011;70(2):309-314. doi:10.1136/ard.2010.132613.

4. Piram M, Koné-Paut I, Lachmann HJ, Frenkel J, Ozen S, Kuemmerle-Deschner J, et al. Validation of the auto-inflammatory diseases activity index (AIDAI) for hereditary recurrent fever syndromes. Ann Rheum Dis. 2014;73(12):2168-2173. doi:10.1136/annrheumdis-2013-203666.

5. Toyofuku E, Takeshita K, Ohnishi H, Kiridoshi Y, Masuoka H, Kadowaki T, et al. Dysregulation of the intestinal microbiome in patients with Haploinsufficiency of A20. Front Cell Infect Microbiol. 2022; 11:787667. doi:10.3389/fcimb.2021.787667.

6. Frémond ML, Rodero MP, Jeremiah N, Belot A, Jeziorski E, Duffy D, et al. Efficacy of the Janus kinase 1/2 inhibitor ruxolitinib in the treatment of vasculopathy associated with TMEM173-activating mutations in 3 children. J Allergy Clin Immunol. 2016;138(6):1752-1755. doi:10.1016/j.jaci.2016.07.015.
