# Supplementary material for: Efficacy and safety of thalidomide in children with monogenic autoinflammatory diseases: a single-center, real-world-evidence study
Source: Pediatr Rheumatol Online J. 2023 Oct 17;21:124. doi: 10.1186/s12969-023-00881-0 (PMC10583446; doi:10.1186/s12969-023-00881-0)
Supplement: Supplementary file 2 — Additional file 2. [file 12969_2023_881_MOESM2_ESM.docx]

Disease distribution and the baseline characteristics of 16 patients

| No. | Diagnosis | Gene Mutation | Age at baseline in years | Gender | Clinical and laboratory manifestations |
| --- | --- | --- | --- | --- | --- |
| 1 | AGS | IFIH1: p. A339D | 4.33 | Male | Rash; growth and intellectual disability; increased liver parenchymal echogenicity; pulmonary interstitial disorders; anaemia; hypothyroidism |
| 2 | AGS | TREX1: p. G47S; p.C154Mfs*3 | 13.58 | Female | Rash; pulmonary interstitial disorders; aortic valve thickening and insufficiency; flexion deformity of the elbow; glaucoma; calcification basal ganglion; cerebral infarction; positive autoantibodies for ANA and anti-dsDNA |
| 3 | AGS | TREX1: p. T22M | 11.50 | Female | Raynaud's phenomenon; xerostomia; thrombocytopenia; positive autoantibodies for ANA, anti-ribonucleoprotein antibody and anti-β2GP1 |
| 4 | Blau syndrome | NOD2: p. R587C | 8.67 | Female | Fever; rash; left atrium enlargement, pericardial effusion; growth retardation; uveitis; |
| 5 | Blau syndrome | NOD2: p.481G>D | 5.0 | Male | Rash; growth retardation; left and right coronary arteries widened and left atrium enlarged; multiple joint swelling; uveitis; delay in anterior fontanelle closure |
| 6 | Blau syndrome | NOD2: p. R334W | 8.75 | Female | Rash; uveitis; anaemia; positive for antimitochondrial antibody |
| 7 | Blau syndrome | NOD2: p. R334W | 10.83 | Female | Rash; left atrial enlargement, hypertension; multiple joint swelling; uveitis |
| 8 | CINCA | NLRP3: p. M664T | 12.0 | Male | Fever; rash; growth and intellectual disability; splenomegaly; pulmonary interstitial disorders; thickening of the pericardium, ventricular dilatation; binaural sensorineural deafness; intracranial calcification, aseptic meningitis; anaemia |
| 9 | CINCA | NLRP3: p. D305N | 11.58 | Female | Rash; growth retardation; bilateral lung translucency decreased; hypertension; arthritis; binaural mixed hearing loss; aseptic meningitis; anaemia |
| 10 | DADA2 | CECR1: p. N85I, p. G284V | 5.1 | Female | Fever; rash; hepatomegaly; cerebral haemorrhage; sequelae of cerebral infarction; anaemia |
| 11 | FMF | MEFV: p. G340R, E148Q, R408Q, P369S | 14.0 | Male | Multiple intestinal ulcers |
| 12 | HA20 | TNFAIP3: p. R271X | 11.50 | Female | Fever; punctate erosion of the rectum; multiple nodules in both lungs; hypothyroidism; positive ANA, anticardiolipin autoantibodies |
| 13 | HA20 | TNFAIP3: p. D70N | 5.75 | Male | Fever; rash |
| 14 | PLAID | PLCG2: p.C1082R | 14.83 | Female | Fever; rash; splenomegaly; interstitial pneumonitis; thickening of the pericardium; anaemia; anti-β2GP1 |
| 15 | SAVI | TMEM173: p. N154S | 2.75 | Male | Fever; rash; growth retardation; pulmonary interstitial disorders, pulmonary bulla; positive ANA, anti-cyclic citrullinated peptide, rheumatoid factor, anti-dsDNA, anti-histone antibody, anti-neutrophilic cytoplasmic autoantibodies |
| 16 | TRAPS | TNFRSF1A: p. T79M | 11.33, | Male | Fever |

ANA, antinuclear antibody; anti- dsDNA, anti-double-stranded; anti-β2GP1, anti-β2 glycoprotein I antibody
